# Gq-Coupled Receptors in Autoimmunity

**DOI:** 10.1155/2016/3969023

**Published:** 2016-01-17

**Authors:** Lu Zhang, Guixiu Shi

**Affiliations:** ^1^Department of Nephrology, The First Affiliated Hospital of Xiamen University, Xiamen 361003, China; ^2^Department of Rheumatology and Clinical Immunology, The First Affiliated Hospital of Xiamen University, Xiamen 361003, China

## Abstract

Heterotrimeric G proteins can be divided into Gi, Gs, Gq/11, and G12/13 subfamilies according to their *α* subunits. The main function of G proteins is transducing signals from G protein coupled receptors (GPCRs), a family of seven transmembrane receptors. In recent years, studies have demonstrated that GPCRs interact with Gq, a member of the Gq/11 subfamily of G proteins. This interaction facilitates the vital role of this family of proteins in immune regulation and autoimmunity, particularly for G*α*q, which is considered the functional *α* subunit of Gq protein. Therefore, understanding the mechanisms through which Gq-coupled receptors control autoreactive lymphocytes is critical and may provide insights into the treatment of autoimmune disorders. In this review, we summarize recent advances in studies of the role of Gq-coupled receptors in autoimmunity, with a focus on their pathologic role and downstream signaling.

## 1. Introduction

Many receptors for hormones, neurotransmitters, neuropeptides, chemokines, and autocrine and paracrine signaling molecules interact with heterotrimeric G proteins to exert their actions on target cells [1]; these receptors are considered G protein coupled receptors (GPCRs) [2]. It is estimated that more than 800 GPCRs are encoded in the mammalian genome [3], supporting that GPCRs are common membrane receptors in cells.

Heterotrimeric G proteins consist of an *α*-subunit, which binds to and hydrolyzes guanosine-5′-triphosphate (GTP), and *β*- and *γ*-subunits, which form an indissociable complex [4]. GPCRs transmit extracellular signals into the cell by binding to and activating different intracellular signaling proteins, termed G proteins (G*αβγ*, families Gi, Gs, Gq/11, G12/13) or arrestins [5]. The Gq proteins, like all heterotrimeric G proteins, are composed of three subunits: G*α*q, G*β*, and G*γ* [6]. G*α*q-GTP and the G*βγ* dimer then transmit receptor-generated signals to downstream effector molecules and protein binding partners until the intrinsic GTPase activity of G*α* hydrolyzes GTP to GDP and the inactive subunits reassociate [6]; this is called the “active and inactive” cycle. Each of the four major subfamilies of G proteins is associated with different signaling pathways: Gq/11 activates the phospholipase C (PLC) family; Gs stimulates the adenylyl cyclase (AC) pathway; Gi/o inhibits AC; and G12/13 activates small GTPases [4].

The Gq/11 subfamily, including Gq, G11, G14, and G15/16, shares structural similarity, and activation of the *α* subunit within each protein complex can activate PLC-*β* [4–7]. Furthermore, all of these four subunits regulate both overlapping and distinct signaling pathways, thereby stimulating inositol lipid (i.e., calcium/protein kinase C (PKC)) signaling through PLC-*β* isoforms [1, 4–9]. Genetic studies using whole animal models have demonstrated the importance of Gq in cardiac, lung, brain, and platelet functions, helping to define the physiological and pathological processes mediated by the Gq [5, 10].

Recent studies have described all four subtypes of Gq/11 coupled GPCRs, including the muscarinic 1, 3, and 5 (M1, M3, and M5) receptors; bombesin receptor, vasopressin receptor, endothelin receptor, thyrotropin-releasing hormone receptor (TRHR), gonadotropin-releasing hormone receptor (GnRHR), membrane estrogen receptor (mER), chemokine receptors, adrenergic receptors (*α*1AR), and angiotensin II type 1 receptor (AT(1)R) [11–13]. In the field of immunology, chemokine and hormone receptors have been shown to function as Gq protein-coupled GPCRs. These GqPCRs are expressed on lymphocytes and are regulated by their ligands in the immune system [14–18]. Abnormal regulation of these receptors may be associated with the pathogenesis of autoimmunity and a variety of autoimmune diseases induced by autoreactive lymphocytes, leading to morbidity and mortality in individuals with autoimmune disorders [19–24].

## 2. The Diversity of Gq-Coupled Receptors in Autoimmunity

Gq is the most commonly studied subclass of the Gq/11 subfamily in the field of immunology [18] and is mainly coupled to sex hormone receptors and some chemokine receptors, which are differentially expressed in certain types of lymphocytes [6, 18] (Table 1).

### 2.1. The GnRHRs and mERs

Gonadotropin-releasing hormone (GnRH) is the primary hormone associated with reproduction; GnRH is known to exert its actions largely through two related Gq/11 protein receptors [25]. The interaction between GnRH and its cognate type I receptor (GnRHR) in the pituitary results in the activation of Gq, PLC-*β*, phospholipase A2 (PLA2), and phospholipase D (PLD) [2]. Sequential activation of phospholipases generates the second messengers inositol 1, 4,5-tris-phosphate (IP3), diacylglycerol (DAG), and arachidonic acid (AA), which are required for Ca^2+^ mobilization. Further activation of various protein kinase C isoforms (PKCs) induces sequential activation of mitogen-activated protein kinases (MAPKs) [26] and promotes nuclear transcription. GnRHR mRNA and protein have been found in the pituitary, lymphocytes, mononuclear cells, and various types of cancer cells [23]. Many autoimmune diseases, particularly systemic lupus erythematosus (SLE), exhibit gender-specific differences, and GnRHRs have been shown to function as immunostimulatory hormone receptors, playing pivotal roles in the observed gender-specific differences in immunity and/or autoimmunity [27].

Acute treatment with GnRH increases the expression of* GnRHR* mRNA in murine thymocytes [28]. Studies in mice and rats have shown that GnRH stimulates the expression of hormone-GqPCR and the interleukin- (IL-) 2 receptor, the proliferation of B and T lymphocytes, and the elevation of serum IgG levels [27]. Jacobson [23] measured* GnRHR* mRNA and GnRH binding in lupus-prone mice after in vivo exposure to GnRH or vehicle. Their results showed that even vehicle-treated females expressed more GnRHR in immune cells than did vehicle-treated males; gender differences were confirmed, with females expressing Gq-coupled hormone receptor mRNA and protein more than males [26]. In mice given GnRH, GnRH (through GqPCR) exacerbated lupus in vivo in females only [27]. Additional studies have shown that GnRH (through GqPCR) stimulates T/B lymphocyte proliferation in vitro in females only [29]. These differences in expression and activation of GnRHR through GqPCR on lymphocytes contribute to the observed gender differences in immunity and/or autoimmunity.

In addition to the pivotal function of intracellular estrogen receptors in autoimmunity, researchers have also shown that mERs can stimulate Gq-coupled GPCRs through PKC and calcium pathways [30]. Rider and Abdou [31] suggested that estrogen acting through Gq-mERs enhances T-cell activation in women with lupus, resulting in amplified T/B-cell interactions, B-cell activation, and autoantibody production.

Thus, the gender differences in GnRH and estrogen production and function can be directly associated with Gq protein receptor expression, which plays a critical role in maintaining the balance of T/B lymphocytes and affects the morbidity of autoimmune diseases that predominantly affect women.

### 2.2. The Chemokine Receptors

Chemokine receptors are expressed on T cells, B cells, monocytes, macrophages, and dendritic cells [32]. Chemokine receptors and their ligand axis play pivotal roles in leukocyte migration, differentiation, adhesion, and activation [32, 33]. Many chemokine receptors have been implicated in the pathogenesis of autoimmune connective tissue diseases such as SLE, rheumatoid arthritis (RA), and systemic sclerosis (SS) [19–21, 32, 34–37]. However, previous studies have demonstrated the indispensable role of chemokine receptors in autoimmune diseases, highlighting the role of Gi protein-coupled chemokine receptors (rather than Gq-coupled receptors) in directing the migration of immune cells, which mostly signal through the canonical AC pathway [19, 36].

In a cell-based study, Arai and Charo [32] showed that monocyte chemotactic protein- (MCP-) 1-related chemokine receptors (mainly CC family receptors) interact with multiple subtypes of G proteins in a cell type-specific manner and that the third intracellular loop of CC type receptors mediates Gq coupling.

Moreover, many studies have shown that chemokine receptors can interact with multiple G-protein subtypes; the coupling is cell type-specific [32]. Shi and colleagues [38] showed that chemokine receptors can be divided into CD38-dependent and -independent subclasses, depending on whether CD38 is needed for the chemotaxis of the ligand. CD38-dependent chemokine receptors couple to Gq, indicating that there is indeed a novel Gq protein-coupled alternative signaling pathway separate from the canonical Gi-coupled classic pathway.

Autoimmunity-associated chemokine receptors mainly include the CC family (CCR5 and CCR7) and the CXC family (CXCR3, CXCR4, CXCR5, and CXCR7) [19, 20, 34, 36–41]. To date, most chemokine receptors, such as CXCR4 and CXCR5 on T cells and B cells, have been shown to be induced by Gi in the classic pathway, while CCR7 and CXCR4 have been shown to be dependent on Gq pathways only on dendritic cells (DCs) [38]. Hence, the dependence of autoimmune diseases on the specific Gq coupled chemokine receptor alone is still unclear. Since this chemokine receptor is engaged and activated in lymphocytes by GPCRs, it is possible that these Gq-coupled receptors may interact functionally with Gi to regulate chemotaxis in lymphocytes during the effector stage [42].

### 2.3. Others

AT(1)R, one of the best-studied GPCRs, signals through Gq to transduce signals on lymphocytes in autoimmune-regulated cardiomyopathy and hypertension [43]. Experimental findings support the concept that the balance between T cell-induced inflammation and T cell suppressor responses is critical for the regulation of blood pressure levels; autoantibodies to these receptors can exacerbate the pathological process [44]. High levels of autoantibodies against the second extracellular loop of *α*1-adrenoceptor (*α*1-AR) are also found in patients with hypertension, suggesting an important role of *α*1-AR and AT(1)R autoimmunity in the pathogenesis and management of hypertension, particularly in patients having high levels of receptor-associated autoantibodies [11, 45, 46]. However, the precise mechanism is still unknown.

## 3. How Do Gq-Coupled Receptors Play a Role in Autoimmunity?

Autoimmunity comprises a variety of autoreactive lymphocytes characterized by the loss of tolerance to a variety of autoantigens and imbalanced humoral and cellular immunity in biological systems [19, 23, 47]. Gq-coupled GPCRs on different lymphocytes can transduce a series of extracellular signals into the nucleus to regulate immune function. Immune responses are coordinated by the extracellular ligand to GPCR on lymphocytes, and activated intracellular Gq protein then activates enzymes, second messengers, protein kinases, and nuclear translocation, consequently inducing the migration, activation, and apoptosis of lymphocytes [48]. This well-orchestrated function of hematopoietic cells is complex; however, it is clear that Gq-coupled receptors and the signaling molecules that reside downstream of these receptors are critical to these functions.

### 3.1. Induction of T-Cell Proliferation by GqPCR

#### 3.1.1. GnRHR

Women are more likely to actively express GnRH and GnRHR than men, as described above, particularly during the reproductive period, at which time immune responses are different [26, 30]. Furthermore, in the spleen and thymus, the expression of G proteins on mononuclear cells differs in a gender-dependent manner [23]. GnRHR exhibits direct immunostimulatory properties, and lymphocytes produce GnRH and express GnRHR [23, 26, 49]. The G protein G*α*q/11 regulates the transduction of signals from multiple hormones from specific cell surface receptors to a variety of intracellular effectors, including the AC pathway, PLC-*β*, and the ion channel pathway [49]. Jacobson et al. [26] demonstrated that antisense nucleotides to G*α*q/11 inhibit hormone and GnRHR signaling, suppressing the proliferation of T cells from female mice after in vitro culture. Additional studies have shown that antisense oligonucleotides to Gq-coupled GPCRs can also be effective in vivo for ameliorating murine lupus; specifically, antisense oligonucleotides directed against G*α*q in female lupus-prone mice effectively reduce serum IgG levels, anti-DNA antibody levels, hematuria, and proteinuria, even in terms of the histopathology of renal biopsies [27]. Thus, these studies demonstrate the utility of GnRH inhibitors to modulate GqPCR activation in mice and suggest a novel potential target for the treatment of lupus.

#### 3.1.2. Chemokine Receptors

Molon et al. [48] have shown that signals mediated by chemokine receptors may compete with T-cell receptor stop signals and determine the duration of T-cell antigen-presenting cell interactions. During T-cell stimulation by antigen-presenting cells, T-cell chemokine receptors coupled to Gq and/or G11 protein are recruited to the immunological synapse. When chemokine receptors are sequestered at the immunological synapse, T cells become insensitive to chemotactic gradients, form more stable conjugates, and enhance proliferation and cytokine production. Thus, chemokine receptor trapping at the immunological synapse enhances T-cell activation by improving T-cell antigen-presenting cell attractions and impeding the “distraction” of successfully engaged T cells by other chemokine sources. Ngai et al. [15] suggested that optimal activation of the T-cell receptor requires signaling through chemokine receptor-Gq and that removal of G*α*q locks cells into a migratory phenotype, making the cell less responsive to T-cell receptor signaling. Previous studies have shown that activation of G*α*q inhibits migration through an Lck-SHP-1 pathway, priming cells for activation through the T-cell receptor-CD3 complex [42]. Thus, these novel GqPCR signaling pathways are involved in mediating the threshold of chemotaxis and T-cell receptor activation, playing an irreplaceable role in immunity and autoimmunity.

### 3.2. Induction of DC and Monocyte Migration by GqPCRs

A Gi-coupled classic pathway activates T lymphocytes and alternative Gq-dependent chemokine receptors, promoting the migration of DCs and monocytes. Furthermore, Gq, similar to CD38, regulates extracellular calcium entry in chemokine-stimulated cells. Gq-deficient (Gnaq−/−) DCs and monocytes are unable to migrate to inflammatory sites and lymph nodes in vivo, demonstrating that this alternative Gq-coupled chemokine receptor signaling pathway is critical for the initiation of immune responses [32, 38].

## 4. The Diversity of Gq-Coupled GPCRs Mediates Activation of Signal-Regulated Pathways

Binding partners to GqPCR distinct from PLC-*β* include novel activators (Ric-8A and tubulin), candidate effectors (RhoGEFs, PI3K, GPCR kinases (GRKs), Btk, and complex regulator of G-protein signaling (RGS) proteins), regulators (RGS proteins and GRKs), and scaffold/adaptor proteins (EBP50/NHERF1, CDP/CD81, caveolin-1, and TPR1) [1, 4, 6, 50]. Downstream of these signaling proteins, signals through GPCR to Gq family members exhibit unexpected differences in signaling pathways and the regulation of gene expression profiles [8, 50].

### 4.1. Gq-Related PLC-*β* and PKC/Calcium Pathways

**Figure 1:**
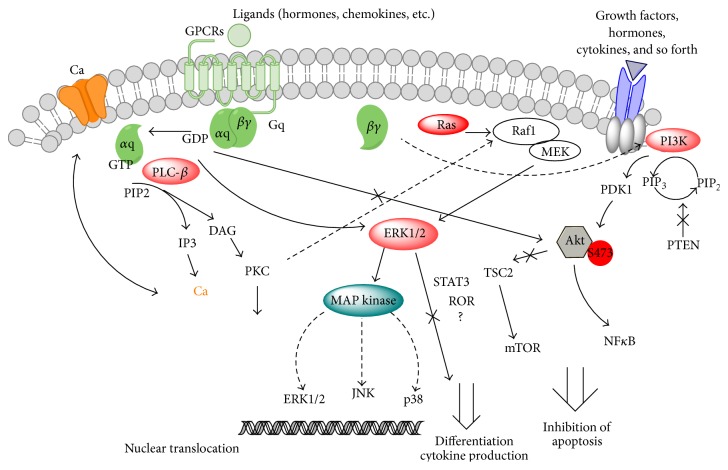
Signaling pathways demonstrating the link between Gq-coupled receptors and induction of autoimmunity. The figure shows the major signaling pathways that are believed to regulate the extracellular signals transduce into lymphocytes, which include the classic PLC-*β*/PKC pathway (left) and inhibition of the PI3K-Akt pathway to maintain normal immune tolerance (right) while Gq can also facilitate the activation of the ERK-MAPK pathway, regulating the differentiation of lymphocytes and controlling the expression of cytokines (middle).

PLC-*β* is the most well-known downstream effector molecule of GqPCR (Figure 1). The canonical pathway for the Gq/11 family is the activation of PLC-*β* enzymes, which catalyze the hydrolysis of the minor membrane phospholipid phosphatidylinositol bisphosphate (PIP2) to release IP3 and DAG [4–7, 13, 14]. These second messengers serve to propagate and amplify the GqPCR-mediated signal with calcium mobilization following release from IP3-regulated intracellular stores and DAG-mediated stimulation of PKC activity [4, 5]. Inositol lipids, DAG, PKC, and calcium each participate in multiple signaling networks, linking Gq family members through a host of different cellular events [1]. This pathway has been widely studied as a marker of GqPCR signaling [8]. As the aforementioned chemokine receptors, there are classic (Gi) and alternative (Gq) coupled GPCR pathways depending on the specific type of the chemokines and chemokine-stimulated cells [38]. The Gi is through AC pathway mentioned in the introduction part. The Gq activates the PLC family that can regulate the extracellular calcium entry in chemokine-stimulated cell and also subsequently influence the downstream effectors such as PI3K/Akt for survival of the cell.

### 4.2. The PI3K-Akt-Mammalian Target of Rapamycin (mTOR) Pathway

Multiple reports have documented the negative influence of Gq-coupled receptors on the growth factor-directed activation of PI3K and Akt isoforms [1, 4–7, 13]. One report showed that G*α*q directly inhibits the PI3K p110a catalytic subunit in vitro [51]. In addition, a previous study also showed that G*α*q represses Akt activation in fibroblast cell lines [52–54] and cardiomyocytes [55, 56]; however, overexpression of G*α*q in cardiomyocytes leads to cardiac hypertrophy and cardiomyocyte apoptosis [10, 57].

PI3K can be activated by the *βγ* dimers released from Gi-coupled receptors [5]. In contrast, Gq normally inhibits PI3K activation and prevents activation of Akt [6, 7, 10, 14, 38]. Furthermore, G*α*q inhibits the activation of the PI3K-Akt pathway, as has been demonstrated in Gn*α*q−/− mice. Indeed, by measurement of the phosphorylation of Akt at Ser473 (phospho-Akt), a phosphorylation site under the control of PI3K demonstrated that the level of phospho-Akt was higher in Gn*α*q−/− mice than in WT B cells [58]. Furthermore, deletion of phosphatase and tensin homolog (PTEN), an inhibitor of PI3K, also promotes mature B-cell survival [59] and can rescue autoreactive B cells from anergy [60]. Interestingly, the autoreactive prone marine zone-like B (MZB) cell compartment is also expanded in mice expressing activated p110 or lacking PTEN [61]. In the absence of G*α*q, B cells constitutively express higher levels of activated Akt and preferentially survive BCR-induced cell death signals and BAFF (B-cell-activating factor of the TNF family, also known as BLyS, for B lymphocyte stimulator) withdrawal in vitro and in vivo [10, 58, 62]. The B cells isolated from multiple models of autoimmunity have been reported to express elevated levels of phospho-Akt [62], and perturbations in the PI3K/Akt axis can lead to the development of autoimmunity [51, 62].

### 4.3. The MAPK/ERK Pathway

In addition to PLC-*β* and PI3K, many studies have demonstrated that Gq-coupled receptors can also regulate other intracellular signaling molecules, such as members of the MAPK family [6, 7, 50, 57]. The MAPK signaling cascade is one of the most ancient and evolutionarily conserved signaling pathways and responds to a broad range of extracellular and intracellular changes [63–67]. Among the MAPKs, p38 MAPK regulates the expression of tumor necrosis factor- (TNF-) *α*, interferon- (IFN-) *γ*, and other cytokines via transcriptional and posttranscriptional mechanisms. Therefore, inhibiting p38 MAPK may abrogate TNF-*α*, providing potential anti-inflammatory effects [65, 68, 69]. Predominant Th1 and Th17 cytokine production are characteristic of many organ-specific autoimmune diseases, and the dysregulation of p38 MAPK activity specifically in autoreactive lymphocytes appears to enhance IL-17 and IFN-*γ* expression [66, 70–72]. Additionally, the ERK pathway can be activated by the small G protein Ras via the Raf group of MAP kinase kinase kinases (MKKKs) [66]. Solid evidence has supported that endothelin-dependent ERK/MAPK activation depends on the GqPCR/PLC-*β*/Ca^2+^/Src signaling cascade [64]. Taken together, these studies have shown that GqPCR and G*α*q are involved in the activation of ERK.

Thus, complex GPCR signaling should be studied as a concerted network at the systems level [73]. The detailed “cross-talk” mechanism between these GqPCR pathways still needs to be explored in the future.

## 5. Perspectives

In this review, we have outlined current evidence supporting the biological, pathological, and cell signaling functions of Gq-coupled GPCRs in autoimmunity. This discussion reinforced the idea of cell signaling diversity and challenged the established paradigm that Gq-coupled GPCR signals in immunology are functionally redundant. Moreover, studies of traditional pathways alone do not account for many Gq-mediated responses; G*α*q-linked signaling suggests that GqPCRs have complex roles in signal transduction that are not yet fully understood.

Our previous studies have shown that Gq is associated with immune diseases and has pivotal roles in autoimmunity [17, 18, 24, 33, 38, 72, 74]. Gq is downregulated in RA's patients and relates to the disease's activity [74]; it can control the RA's progress via Th17 differentiation [72]. While Gq-containing G proteins can regulate B-cell selection and survival and are required to prevent B-cell-dependent autoimmunity [38], the deficiency of Gq can enhance the T-cell's survival [17] and influence the migration of DCs and neutrophils. Based on our unpublished data it is also related to autoinflammatory diseases. Therefore, we are interested in clarifying the role of Gq-coupled GPCRs in immune tolerance and autoimmunity, with the aim of improving therapeutic approached. Nonetheless, there are still some limitations to the available data describing the role of Gq-coupled GPCRs in autoreactive lymphocytes. Further studies using in vitro-derived lymphocytes may not accurately reflect the situations occurring in vivo. Moreover, the aforementioned studies involved different races and small patient populations, which may also have influenced the final results.

To date, many studies have focused on Gq-coupled membrane receptors and, to a lesser extent, G11. However, relatively little is known about G14 and G15/16. Future studies of autoimmunity may improve our understanding of the unique cell signaling roles and properties of other Gq/11 family proteins, including G14 and G15/16. Taken together, our discussion herein summarizes our current understanding of the complexity of Gq-coupled membrane receptor signaling and highlights many exciting new areas for future investigations in autoimmunity.

## Figures and Tables

**Table 1:** Gq-coupled GPCRs in autoimmunity.

Type	AID	Cell type	Disease model	Function in general	References
GnRHR	SLE	Lymphocytes Mononuclear cells Cancer cells	Lupus-prone mice	Through high level of GnRH stimulates the expression of hormone-GqPCR and the interleukin- (IL-) 2 receptor, the proliferation of B and T lymphocytes, and the elevation of serum IgG levels	[23, 26–29]

mER	SLE	T/B lymphocytes	Lupus patients	Amplify T/B-cell interactions, B-cell activation, and autoantibody production	[30, 31]

Chemokine receptor	SLE RA SS	T lymphocytes DCs Monocytes Neutrophils (CD38-dependent)	Gn*α*q−/− mice	(1) Compete with T-cell receptor stop signals and determine the duration of T-cell-APC interactions, form more stable conjugates, and enhance proliferation and cytokine production (2) DCs and monocytes' migration to inflammatory sites and lymph nodes	[15, 19–21, 32, 38, 42, 48]

AT(1)R	Autoimmune-regulated cardiomyopathy and HTN	T lymphocytes	Gq TG mice	Unbalance between T-cell-induced inflammation and T-cell suppressor responses for the regulation of pathological process	[43, 44]

*α*1-AR	HTN	Lymphocytes	HTN patients	High levels of autoantibodies against the second extracellular loop of *α*1-adrenoceptor (*α*1-AR) in patients with hypertension	[11, 45, 46]

AID: autoimmune disease; HTN: hypertension; APC: antigen-presenting cell.
